# IMPROVING OUTCOMES IN OLDER VETERANS WITH HEART FAILURE AND CKD: FROM PROPENSITY SCORE USE TO AI

**DOI:** 10.1093/geroni/igae098.0938

**Published:** 2024-12-31

**Authors:** Ali Ahmed

**Affiliations:** Washington DC Veterans Medical Center, VA Medical Center, District of Columbia, United States

## Abstract

Older adults are often excluded from randomized controlled trials (RCT). Consequently, gold-standard RCT-evidence to improve outcomes in older adults is often unavailable. Properly-designed outcome-blinded propensity score matched studies can provide high-quality non-RCT evidence when RCTs are impractical, unethical, or lack sponsors. Renin-angiotensin system inhibitors (RASIs) improve outcomes in patients with heart failure (HF) and chronic kidney disease (CKD). National HF guidelines recommend that these drugs be prescribed in higher doses, but be used with caution in those with CKD as it may worsen kidney function. Dr. Ahmed has shown that RASIs improve survival in older patients with HF and CKD. However, concerns remain about their effect on kidneys. Recent findings from VA-Merit Review grant suggests that high-dose RASIs lower risk of kidney failure and death in octogenarians with HF and CKD. Additionally, artificial intelligence methods can be utilized to personalize care for this heterogeneous and diverse population of older patients.

